# Prevalence of Merkel Cell Polyomavirus in Normal and Lesional Skin: A Systematic Review and Meta-Analysis

**DOI:** 10.3389/fonc.2022.868781

**Published:** 2022-03-22

**Authors:** Wilson A. Wijaya, Yu Liu, Yong Qing, Zhengyong Li

**Affiliations:** Department of Burn and Plastic Surgery, West China Hospital, Sichuan University, Chengdu, China

**Keywords:** merkel cell carcinoma, merkel cell polyomavirus, prevalence, infectivity, pathogenesis, skin cancer

## Abstract

The prevalence of Merkel cell polyomavirus(MCPyV) in Merkel cell carcinoma(MCC) and non-MCC skin lesions and its possible role in the etiology of other skin diseases remain controversial. To systematically assess the association between MCPyV infection and MCC, non-MCC skin lesions, and normal skin. For this systematic review and meta-analysis, a comprehensive search for eligible studies was conducted using Medline Ovid, Pubmed, Web of Science, and the Cochrane CENTRAL databases until August 2021; references were searched to identify additional studies. Observational studies that investigated the association between MCPyV infection and MCC, non-MCC skin lesions, and normal skin using polymerase chain reaction(PCR) as a detection method and provided sufficient data to calculate the prevalence of MCPyV positivity. A total of 50 articles were included in the study after exclusion criteria were applied. Two reviewers independently reviewed and assessed the eligibility of the studies, and all disagreements were resolved by consensus. To determine the association between MCPyV and MCC, overall odds ratio (OR) were calculated with 95% CI using a random-effects model. Single-arm meta-analyses were performed to examine the prevalence rate of MCPyV+ in MCC, non-MCC skin lesions, and normal skin. The primary analysis was the prevalence rate of MCPyV+ in MCC. Secondary outcomes included the prevalence rate of MCPyV+ in non-MCC skin lesions and normal skin. A total of 50 studies involving 5428 patients were reviewed based on our inclusion and exclusion criteria. Compared with the control group, MCPyV infection was significantly associated with MCC (OR = 3.51, 95% CI = 2.96 - 4.05). The global prevalence of MCPyV+ in MCC, melanoma, squamous cell carcinoma, basal cell carcinoma, Bowen’s disease, actinic keratosis, keratoacanthoma, seborrheic keratosis, and normal skin was 80%, 4%, 15%, 15%, 21%, 6%, 20%, 10%, and 11%, respectively. The current results suggest that MCPyV infection is significantly associated with an increased risk of MCC. However, the low prevalence rate of MCPyV+ in non-MCC skin lesions does not exclude a pathogenic association of this virus with the development of non-MCC skin lesions.

## Introduction

Merkel cell carcinoma(MCC) is a rare, high-grade, aggressive cutaneous neuroendocrine tumor originally discovered in 1972 (1–3). MCC is prone to recurrence, regional metastases that frequently recur in lymph nodes, and distant metastases. Advanced age(> 50 years), demographic characteristics(predominantly European) and sun-exposed skin(ultraviolet radiation) are established risk factors for MCC (4, 5). In recent decades, the incidence of MCC has increased, as has the mortality rate (6).

Polyomaviruses(PyVs) are small, double-stranded DNA-based viruses that are usually non- oncogenic for their hosts but may be oncogenic to some species under certain circumstances (7). PyVs have three major genomic regions: an early region encoding large T antigen (LTA) and small T antigen (STA), both viral oncoproteins with replicative functions; a late region encoding viral structural proteins such as VP1, VP2, and VP3; and a noncoding control region(NCCR) that controls viral replication (8, 9). The identification of Merkel cell polyomavirus(MCPyV) by digital transcriptome analysis was a significant leap in the knowledge of the pathogenesis of MCC (8). According to molecular epidemiological studies, MCPyV has a wide range of prevalences in MCC. The prevalence of MCPyV varies widely worldwide, ranging from approximately 25% in Australian MCC patients to 100% in a French study (10, 11). In addition, MCPyV DNA has also been detected in non-MCC skin lesions and normal skin (12, 13). However, the mechanism of MCPyV infection and the prevalence of MCPyV in non-MCC skin lesions and its potential role in the pathogenesis of other malignant skin diseases are still unknown. To better understand this problem, we performed a systematic review and meta-analysis to examine the relationship between MCPyV and MCC, non-MCC skin lesions, and normal skin.

## Methods

### Literature Search

This article complies with the Declaration of Helsinki. Preferred Reporting Items for Systematic Reviews and Meta-analyses(PRISMA) guideline was used to conduct the study. Two of us(WAW and LY) comprehensively searched Medline Ovid, Pubmed, Web of Science, and the Cochrane CENTRAL databases from inception to August 1, 2021. Search terms were “merkel cell polyomavirus” and “skin neoplasms,” “skin malignancy,” “skin cancer,” “merkel cell carcinoma,” “squamous cell carcinoma,” “basal cell carcinoma,” “melanoma,” “bowen disease,” “actinic keratosis,” “keratoacanthoma,” “seborrheic keratosis” “non-lesional skin” or “normal skin.” Searches were limited to human participants and English-language publications. We also conducted manual searches of the reference lists of the extracted articles to identify additional relevant publications. Only studies meeting the eligibility criteria outlined below were included in the meta-analysis.

### Eligibility Criteria

The extracted data were required to meet the following criteria: (1) designed as a cohort, case-control study, or cross-sectional study; (2) confirmed the presence of MCPyV by polymerase chain reaction(PCR); (3) reported the detection of MCPyV in MCC, squamous cell carcinoma(SCC), basal cell carcinoma(BCC), melanoma, Bowen’s disease, actinic keratosis, keratoacanthoma, seborrheic keratosis or normal skin; (4) full text available.

Studies that met more than one of the following criteria were excluded: (1) insufficient raw data to estimate the outcome; (2) animal study, *in vitro* study, case report, review, editorial, or commentary; (2) the available data could not be extracted from the article by calculation or by contacting the authors; and (3) multiple studies with overlapping samples. The studies with a more significant number of patients were selected when overlapping study samples were identified. Two reviewers(WAW and LY) independently performed the study selection process, and consensus resolved disagreements.

### Data Extraction and Quality Assessment

Data were extracted by the two independent reviewers (WAW and LY) using a structured Excel(Microsoft Corp., Redmond, Washington) data collection spreadsheet as a priori. Discrepancies were discussed and resolved within the research team. The following data were retrieved for the included studies: first author, publication year, country, study design, number of patients in each group (MCC, SCC, BCC, melanoma, Bowen’s disease, actinic keratosis, keratoacanthoma, seborrheic keratosis, and normal skin), number of patients in each group above with MCPyV+, sample types [frozen section(FR) or formalin-fixed paraffin-embedded (FFPE)], PCR primers, and immune status. Eligible studies were further divided into two different analyses: primary and secondary. The primary analysis was the prevalence rate of MCPyV in MCC. Secondary outcomes included the prevalence rate of MCPyV in non-MCC skin lesions(melanoma, SCC, BCC, Bowen’s disease, actinic keratosis, keratoacanthoma, and seborrheic keratosis) and normal skin.

Quality assessment of included studies was performed using the Newcastle-Ottawa scale for cohort and case-control studies (14). The Newcastle-Ottawa scale consists of selection, comparability, and outcome(or exposure for case-control studies). A study can receive one score in each of the domains of selection and outcome and two scores in the domain of comparability. Studies with a low risk of bias had a score of less than 4, those with a score of 4 to 6 had an intermediate risk of bias, and those with a score of 7 or higher had a low risk of bias.

### Statistical Analysis

Stata 15.1(StataCorp, College Station, TX USA, 2018) was used to analyze the data after it had been checked for consistency. The “metaprop” command was used to generate pooled effect size(ES) for noncomparative binary outcomes. The 95% confidence interval (CI) was generated using the DerSimonian-Laird random-effects model with FreemanTukey double arcsine transformation and evaluated using the Wilson score technique. The Cochran Q and I^2^ statistics were used to test for heterogeneity among the chosen studies. Mild, moderate, and severe heterogeneity were defined as I^2^ statistics of 25% - 50%, 50% - 75%, and >75%, respectively. A random-effects model was used to produce the pooled estimate and 95% CI if heterogeneity was more than 50%. The Mantel-Haenszel method was used to evaluate dichotomous variables, and the results are presented as ORs. Subgroup analysis and meta-regression were employed when heterogeneity was evident based on important variables(country, continent, sample type). Sensitivity analysis was performed to estimate the influence of a single study on the pooled ORs. Statistical significance is defined as a two-tailed P-value of less than 0.05. The visual estimation of a funnel pot, Egger’s test, Begg’s test, and the trim & fill method were used to determine and correct publication bias (P =0.05 was considered significant).

## Results

### Search Results and Included Trials

**Figure 1:**
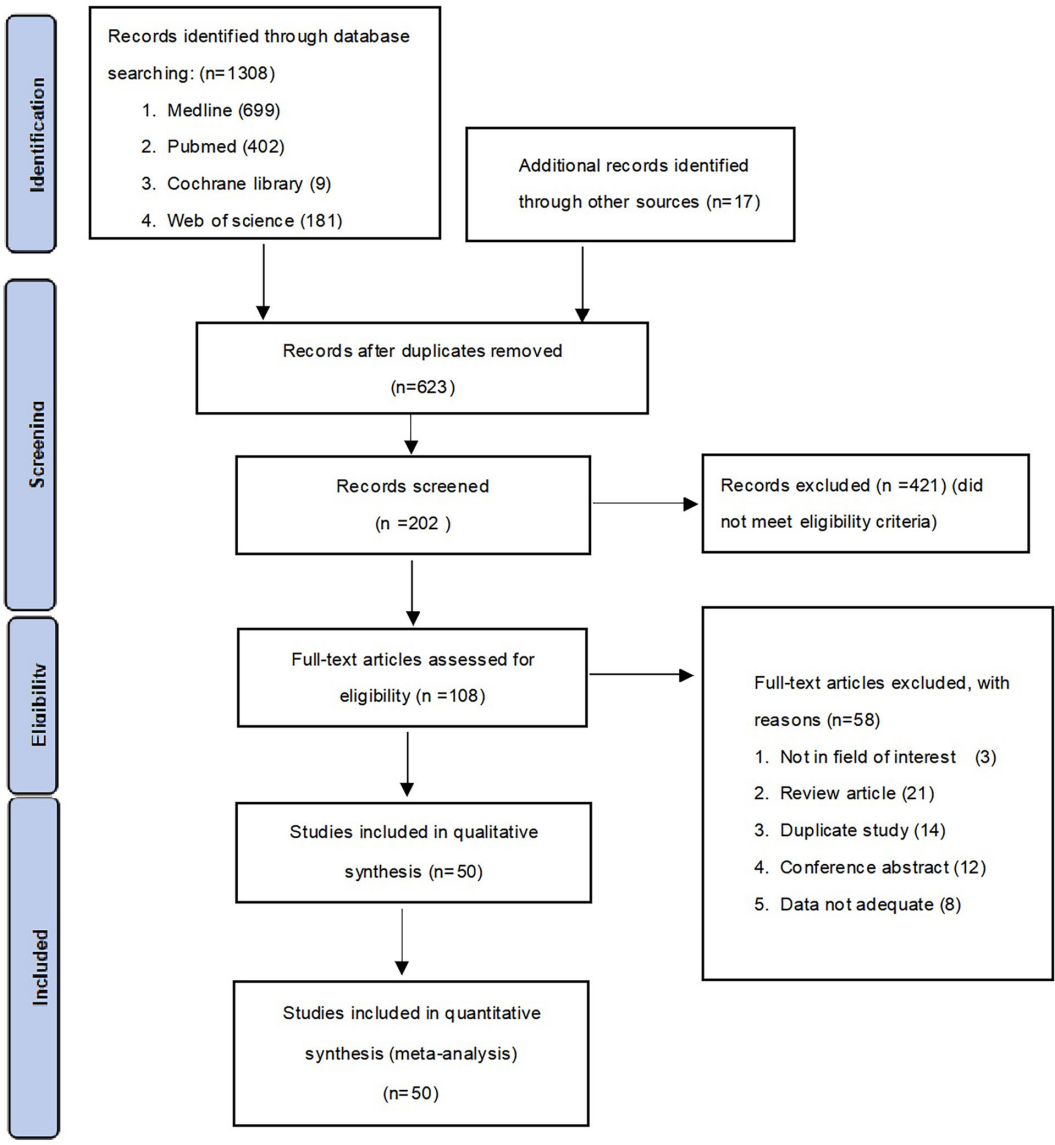
Flow diagram of studies selection.

A total of 1308 studies were identified through the literature search. After adjustment for duplicates, 623 articles remained. Of these, 421 articles were removed after reviewing the titles and abstracts. After a full-text review of the remaining 108 articles, 58 articles were further excluded based on the following criteria: 3 studies were not in the field of interest, 21 studies were review articles, 14 studies were duplicates, 12 were conference abstracts, and eight studies had insufficient data. Finally, 50 studies consisting of 31 case-control studies (1812 participants) and 19 cross-sectional studies (3616 participants) were included in the meta-analysis. The flowchart for the selection process and detailed identification is shown in **Figure 1**. The 50 included studies were published between 2008 and 2021 in 15 different countries. Thirty five studies reported the prevalence of MCPyV+ in MCC patients, 13 studies in normal skin, 11 studies in cutaneous melanoma patients, 23 studies in SCC patients, 17 studies in BCC patients, seven studies in keratoacanthoma patients, six studies in Bowen’s disease and actinic keratosis patients, and five studies in patients with seborrheic keratosis. Thirty studies (8, 10, 11, 13, 15–40) received a score of 7 on the NOS score, while 1 study (41) received a score of 6. All were classified as low risk of bias after quality assessment. However, 19 studies (42–60) had a intermediate risk of bias. **Table 1** summarizes the characteristics of the included articles, and the quality of the papers is assessed in **Table S1**.

**Table 1:** Main characteristics of included studies.

Study	Country	Study type	Sample types	MCC	Control	Normal skin	Melanoma	SCC	BCC	Bowen	Actinic keratosis	Keratoacanthoma	Seborrheic keratoses	PCR primers	Immune status
Feng et al. 2008 (8)	USA	case control	Fr	10	84	NR	NR	NR	NR	NR	NR	NR	NR	LT1, LT3, VP1	control group: skin and skin tumor from 25 immunocompetent and immunosuppressed without MCC; MCC: NR
Kassem et al. 2008 (15)	Germany	case control	FFPE	39	45	NR	NR	NR	NR	NR	NR	NR	NR	LT1, LT3, VP1 , M1/2	NR
Becker et al. 2009 (16)	Germany	case control	FFPE	53	24	NR	NR	NR	24	NR	NR	NR	NR	LT1, LT3	NR
Garneski et al. 2009 (10)	USA / Australia	case control	NR	37	30	15	NR	15	NR	NR	NR	NR	NR	LT1	NR
Helmbold et al. 2009 (17)	Germany	case control	FFPE	98	44	NR	NR	NR	NR	NR	NR	NR	NR	MCPyV	NR
Kassem et al. 2009 (42)	Germany	cross-sectional	FFPE	NR	NMSC immunosuppresed: 56; immunocompetent.: 147	NR	NR	SCC immunosuppresed: /25; immunocompetent:28	BCC immunosuppresed:18; immunocompetent:96	Bowen immunosuppresed:13; immunocompetent :24	NR	NR	NR	LT3, VP1	56 NMSC immunosuppressed ; 147 NMSC immunocompetent
Sihto et al. 2009 (18)	Finland	case control	FFPE	114	22	NR	7	NR	NR	NR	NR	NR	NR	LTA	Eight MCC subjects were considered to be immunocompromised, of which five of them were positive for MCPyV
Varga et al. 2009 (19)	Hungary	case control	FFPE	7	29	NR	3	13	10	NR	NR	NR	NR	LT1, LT3, VP1	NR
Touze et al. 2009 (20)	France	case control	FFPE/Fr	32	9	NR	NR	NR	NR	NR	NR	NR	NR	LT1, VP1	NR
Wieland et al. 2009 (21)	Germany	case control	NR	34	95	34	12	6	21	4	7	7	NR	LT1, LT3	immunocompetent
Andres et al. 2009 (22)	Germany	case control	FFPE	33	33	NR	10	NR	11	NR	NR	NR	12	LTA (MCV 138), STA (MCV 191)	NR
Bhatia et al. 2009 (23)	USA	case control	FFPE	23	52	NR	NR	NR	NR	NR	NR	NR	NR	MCPyV (EU375804)	NR
Dworkin et al. 2009 (43)	USA	cross-sectional	FFPE	NR	NR	63	NR	177	NR	NR	NR	NR	NR	LT3, VP1	immunocompetent and immunosuppressed
Foulongne et al. 2009 (24)	France	case control	FFPE/Fr	11	24	6	NR	NR	NR	NR	NR	NR	NR	LT1, LT3, VP1	NR
Sastre-Garau et al. 2009 (11)	France	case control	FFPE/Fr	10	1241	4	13	NR	13	NR	NR	NR	NR	MCV350 (MCV_ST_A, MCV_ST_B, MCV_LT_C, MCV_LT_D,MCV_VP1_A,MCV_VP1_B)	
Mertz et al. 2010 (44)	Switzerland	cross-sectional	FFPE	NR	NR	NR	NR	6	3	8	4	3	3	LT1, LT3, VP1	immunocompromised
Loyo et al. 2010 (13)	USA	case control	FFPE/Fr	7	286	9	NR	12	NR	NR	NR	NR	NR	LT3, VP1	NR
Mangana et al. 2010 (25)	Switzerland	case control	FFPE	30	19	11	NR	8	NR	NR	NR	NR	NR	LT1, LT3, VP1	NR
Jung et al. 2011 (26)	Korea	case control	FFPE	11	24	NR	12	NR	NR	NR	NR	NR	NR	LT1, LT3, VP1 LT1-1, LT1-1a, LT3a	NR
Kuwamoto et al. 2011 (41)	Japan	case control	FFPE	22	3	NR	1	1	NR	NR	NR	NR	NR	LT3, MCVPS1, MCVKW3	NR
Murakami et al. 2011 (45)	Japan	cross-sectional	FFPE	NR	NR	NR	NR	30	10	NR	NR	NR	NR	LT3, VP1	immunocompetent
Martel-Jantin et al. 2012 (27)	France	case control	FFPE/Fr	36	31	NR	NR	NR	NR	NR	NR	NR	NR	LT3, MerkT	NR
Ota et al. 2012 (46)	Japan	cross-sectional	FFPE	9	142	NR	5	NR	46	34	52	NR	5	ST, LT1, LT2, VP1, VP2, VP3	1 case in which primary MCC had developed within 2 months after a living donor liver transplantation for fulminant hepatitis of unknown etiology
Rodig et al. 2012 (28)	USA	case control	FFPE	51	6	NR	NR	NR	NR	NR	NR	NR	NR	LT2, Set 6, 7, 9 LT3	14 MCC patients had a prior history, comorbidity, or medication consistent with an immunocompromised state from malignancy, autoimmune disease, or solid organ transplantation.
Rollison et al. 2012 (47)	USA	cross-sectional	Fr	NR	NR	NR	NR	145	NR	NR	NR	NR	NR	LTA, VP1F	9 SCC cases reported a history of organ transplantation.
Scola et al. 2012 (48)	Germany	cross-sectional	FFPE	NR	NR	NR	NR	52	41	8	31	42	NR	LTA	immunocompetent
Wieland et al. 2012 (49)	Germany	cross-sectional	FFPE	43	NR	NR	NR	52	NR	NR	NR	42	NR	LTA	immunocompetent
Iwasaki et al. 2013 (50)	Japan	cross-sectional	FFPE	39	NR	NR	NR	NR	NR	NR	NR	NR	NR	ST, LT3, VP1	NR
Chun et al. 2013 (29)	Korea	case control	FFPE	7	32	NR	NR	8	8	NR	8	NR	8	LTA (MCV 138), STA (MCV 191)	immunocompetent
Hattori et al. 2013 (30)	Japan	case control	FFPE/Fr	26	21	NR	NR	11	10	NR	NR	NR	NR	LT1, LT3, VP1	NR
Fukumoto et al. 2013 (31)	Japan	case control	FFPE/Fr	30	183	NR	NR	NR	NR	NR	NR	NR	NR	STA, LT1,LT3, VP1, VP2, VP3	immunocompetent and immunosuppressed
Imajoh et al. 2013 (51)	Japan	cross-sectional	FFPE	NR	NR	NR	47	63	50	NR	NR	NR	NR	MCPyV (EU375804)	NR
Mertz et al. 2013 (52)	Switzerland	cross-sectional	FFPE	NR	NR	47	NR	immunocompetent:60; immunosuppresed: 15	immunocompetent : 71; immunosuppresed:17	NR	NR	NR	NR	LTA (DTS1, DTS2)	immunocompetent and immunosuppressed
Leroux-Kozal et al. 2015 (32)	France	case control	FFPE/Fr	36	3	NR	NR	NR	NR	NR	NR	NR	NR	LTA	immunocompetent
Falchook et al. 2015 (53)	USA	cross-sectional	FFPE	NR	NR	9	NR	12	NR	NR	NR	NR	NR	STA	NR
Bellot et al. 2016 (54)	Brazil	cross-sectional	Fr	immunosuppressed : 1	immunosuppressed: 5; immunocompetent: 86	NR	NR	11	65; immunosuppressed : 5	4	5	1	NR	LT3	A total of five tumours were extracted from two immuno suppressed patients [two BCCs from a kidney trans plant patient, and two BCCs and one MCC from a patient infected with human immunodeficiency virus (HIV)
Haeggblom et al. 2016 (55)	Sweden	cross-sectional	FFPE	NR	NR	NR	NR	NR	NR	NR	NR	22	NR	LTA, VP1	NR
Alvarez-Arguelles et al. 2017 (33)	Spain	case control	FFPE	34	6	NR	NR	NR	NR	NR	NR	NR	NR	VP1	Five MCC patients were immunocompromised
Arvia et al. 2017 (56)	Italy	cross-sectional	FFPE	12	64	10	NR	34	NR	NR	NR	NR	NR	MCPyV	NR
Wang et al. 2017 (34)	USA	case control	FFPE	52	19	18	NR	NR	NR	NR	NR	NR	NR	MCPyV LTAg (LT2) and sTAg (SET9)	NR
Mohebbi et al. 2017 (35)	Iran	case control	FFPE	NR	NR	50	NR	50	NR	NR	NR	NR	NR	LT3, VP1	NR
Kervarrec et al. 2018 (36)	France	case control	FFPE	99	12	NR	NR	NR	NR	NR	NR	NR	NR	MCPyV VP1, LTA	immunocompetent and immunosuppressed
Hillen et al. 2018 (37)	Germany	case control	FFPE	NR	NR	16	NR	NR	NR	NR	NR	NR	23	VP1, M1M2	NR
Kim et al. 2019 (57)	Korea	cross-sectional	FFPE	NR	NR	NR	NR	NR	NR	NR	NR	10	NR	STA, LTA (MCP138, MCP191)	immunocompetent
Rekhi et al. 2019 (58)	India	cross-sectional	FFPE	12	NR	NR	NR	NR	NR	NR	NR	NR	NR	STA, LTA	NR
Neto et al. 2019 (38)	Brazil	case control	FFPE	13	20	NR	NR	NR	NR	NR	NR	NR	NR	LT1	NR
Goncalves et al. 2020 (59)	Brazil	cross-sectional	Fr	NR	NR	NR	NR	NR	35	NR	NR	NR	NR	LT3	immunocompetent and immunosuppressed
Costa et al. 2020 (60)	Brazil	cross-sectional	FFPE	20	89	NR	14	20	20	NR	NR	NR	NR	LT1	18 patients were immunosuppressed
Mokanszki et al. 2021 (39)	Hungary	case control	FFPE	9	60	NR	60	NR	NR	NR	NR	NR	NR	LT1, LT3, VP1	NR
Motavalli et al. 2021 (40)	Iran	case control	NR	NR	NR	NR	NR	20	60	NR	NR	NR	NR	LT3, VP1	immunocompetent

Fr, Frozen section; FFPE, formalin-fixed paraffin-embedded material; NR, Not Reported; MCC, Merkel Cell Carcinoma; SCC, Squamous Cell Carcinoma; BCC, Basal Cell Carcinoma; NMSC, Non-Melanoma Skin Cancer; LTA, Large T antigen; STA, Small T antigen; PCR, Polymerase Chain Reaction.

### Primary Meta-Analysis: Merkel Cell Polyomavirus Prevalence in MCC

**Figure 2:**
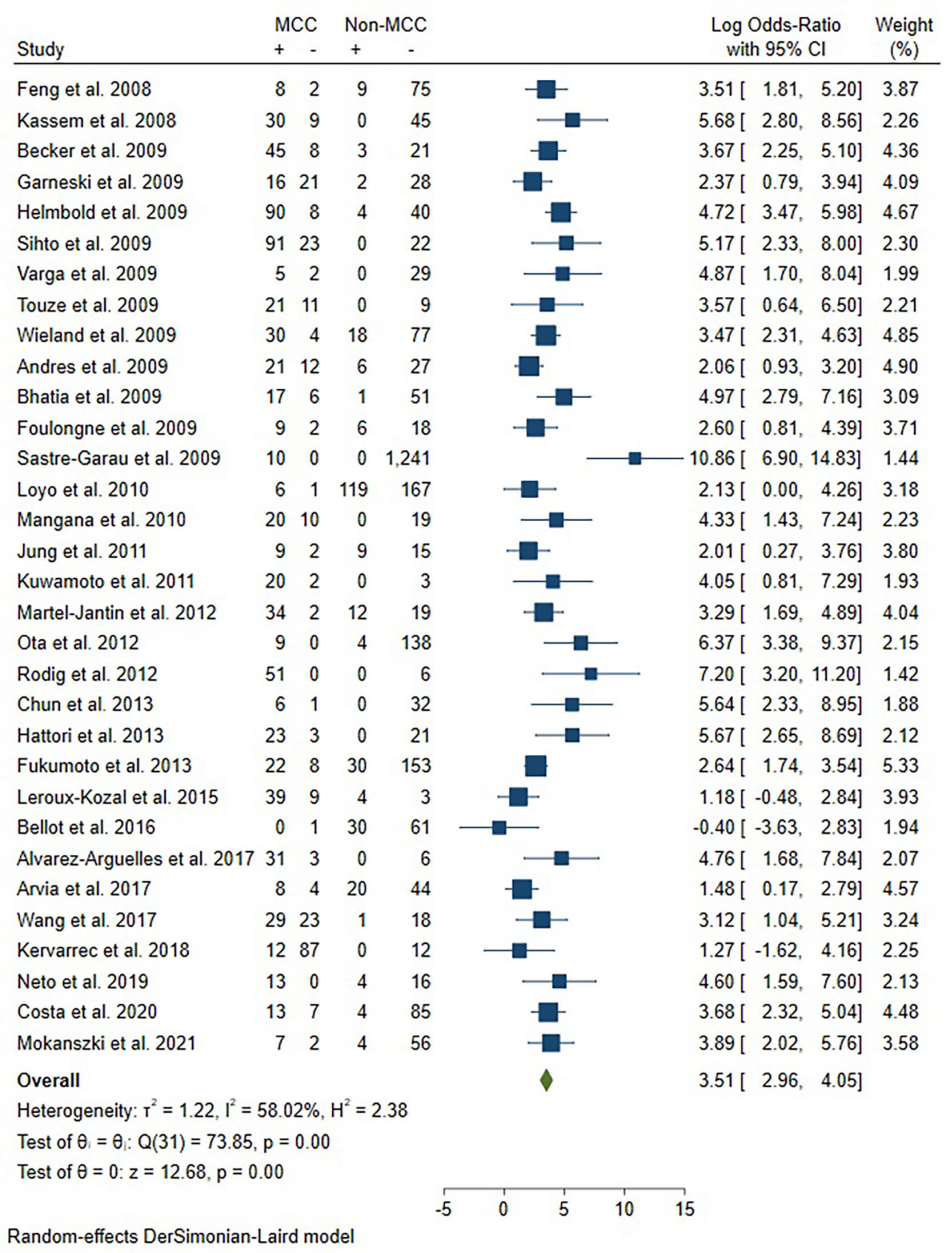
Forest plot illustrating the odd ratio for the association between MCPyV and MCC.

In the pooled analysis, the association between MCPyV and MCC was significant with an adjusted pooled OR of 3.51 (95% CI = 2.96 - 4.05, *P*<0.05) in the random-effects model due to significant heterogeneity between studies (I^2^ = 58.02%)(**Figure 2**). The meta-regression analysis revealed that country (*P*=0.474), continent (*P*=0.220) and sample type (*P*=0.675) did not influence the heterogeneity between studies. The sensitivity analysis showed that no single study influenced the recalculated pooled ORs **(** **Figure S1** **)**. Visual inspection of the funnel plot showed evidence of publication bias (**Figure S2**), which was confirmed by Egger’s test(*P*= 0.0006) and Begg’s test(*P*= 0.0037). We then applied the trim and fill method to correct the asymmetry of the funnel plot (**Figure S3**). Pooled analysis included the imputed studies continued to indicate a statistically significant association between MCPyV and MCC. The result showed that the effect of publication bias was not significant and the conclusion was relatively stable.

**Figure 3:**
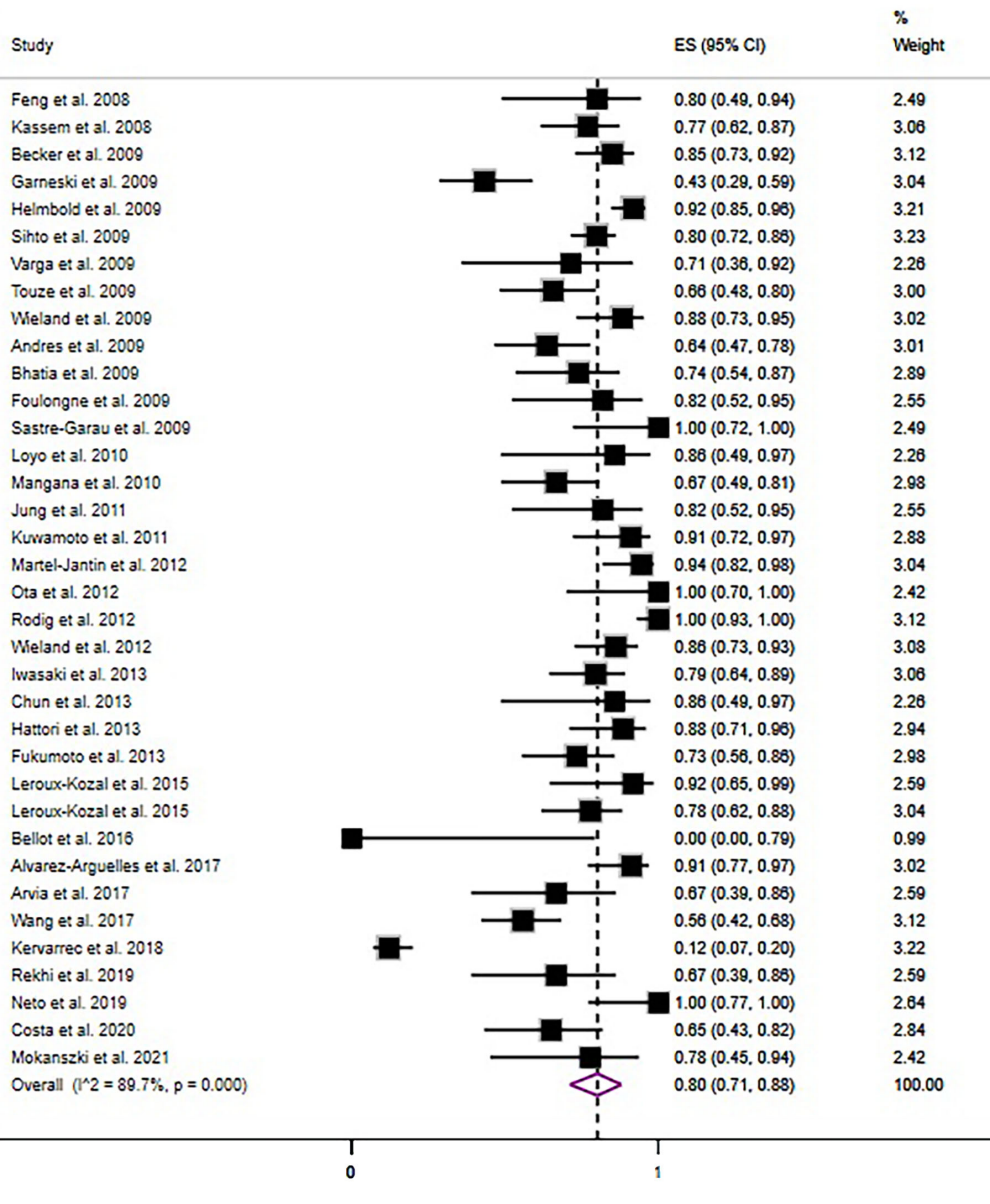
Forest plot illustrating the pooled prevalence rate of the MCPyV positivity in MCC.

The overall pooled prevalence rate of MCPyV+ in MCC was 80% (95% CI = 71% - 88%, I^2 =^ 89.93%, *P*<0.05)(**Figure 3**). We then performed a subgroup analysis based on country, continent, and sample type (frozen section or formalin-fixed paraffin-embedded material). This pooled rate remained consistent in the subgroup analysis, with statistically significant heterogeneity between subgroups (**Table 2** and **Figures S4–6**). There was no obvious source of heterogeneity in the meta-regression analysis(*P*=0.587). The funnel plot, Egger’s test (*P* = 0.284) and Begg’s test (*P* = 0.173) did not indicate publication bias.

**Table 2:** Subgroup results for MCC.

	Stratification criterion	Number of studies	Pooled results (95% CI)	I^2^	P-value for difference
**Merkel cell carcinoma**	**Overall**	35	80% (71% - 88%)	89.93%	<0.05
	**Country**				
	- USA	5	83% (53% - 100%)	91.5%	<0.05
	- Germany	6	83% (75% - 90%)	65.9%	<0.05
	- USA/Australia	1	43% (29% - 59%)	-	-
	- Finland	1	80% (72% - 86%)	-	-
	- Hungary	2	75% (49% - 95%)	-	-
	- France	6	75% (36% - 99%)	96.8%	<0.05
	- Switzerland	1	67% (49% - 81%)	-	-
	- Korea	2	84% (61% - 98%)	-	-
	- Japan	5	86% (76% - 93%)	34.8%	0.19
	- Brazil	3	80% (21% - 100%)	-	-
	- Spain	1	91% (77% - 97%)	-	-
	- Italy	1	67% (39% - 86%)	-	-
	- India	1	67% (39% - 86%)	-	-
	**Continent**				
	- America	9	78% (54% - 96%)	89.9%	<0.05
	- Europe	18	78% (65% - 89%)	93.1%	<0.05
	- Asia	8	84% (76% - 90%)	13.3%	0.33
	**Sample types**				
	- Frozen section (Fr)	3	85% (46% - 100%)	-	-
	- Formalin-fixed paraffin-embedded (FFPE)	24	79% (67% - 89%)	92.2%	<0.05
	- Formalin-fixed paraffin-embedded / Frozen section (FFPE/Fr)	7	85% (74% - 94%)	59.8%	0.02
	- Not reported (NR)	2	67% (55% - 77%)	-	-
**Normal skin**	**Overall**	13	11% (4% - 20%)	71.2%	<0.05
	**Country**				
	- USA	4	19% (1%- 48%)	84.2%	<0.05
	- Germany	2	22% (11% - 35%)	-	-
	- USA/Australia	1	0% (0-20%)	-	-
	- France	2	7% (0%-37%)	-	-
	- Switzerland	2	15% (6% - 26%)	-	-
	- Italy	1	10% (2% - 40%)	-	-
	- Iran	1	2% (0% - 10%)	-	-
	**Continent**				
	- America	5	13% (0% - 36%)	83.5%	<0.05
	- Europe	7	15% (9% - 23%)	3.3%	0.40
	- Asia	1	2% (0% - 10%)	-	-
	**Sample type**				
	- Formalin-fixed paraffin-embedded (FFPE)	8	8% (3% - 16%)	57.9%	0.02
	- Formalin-fixed paraffin-embedded / Frozen section (FFPE/Fr)	3	30% (0% - 84%)	-	-
	- Not reported (NR)	2	13% (5% - 25%)	-	-
**Melanoma**	**Overall**	11	4% (1% - 9%)	0%	0.478
	**Country**				
	- Finland	1	0%(0% - 35%)	-	-
	- Hungary	2	2% (0% - 10%)	-	-
	- Germany	2	13% (1% - 32%)	-	-
	- France	1	0% (0% - 23%)	-	-
	- Korea	1	0% (0% - 24%)	-	-
	- Japan	3	8% (0% - 22%)	-	-
	- Brazil	1	7% (1% - 31%)	-	-
	**Continent**				
	- America	1	7% (1% - 31%)	-	-
	- Europe	6	3% (0% - 9%)	0%	0.66
	- Asia	4	2% (0% - 18%)	36.8%	0.19
	**Sample type**				
	- Formalin-fixed paraffin-embedded (FFPE)	9	4% (0% - 9%)	0%	0.55
	- Formalin-fixed paraffin-embedded / Frozen section (FFPE/Fr)	1	0% (0% - 23%)	-	-
	- Not reported (NR)	1	17% (5% - 45%)	-	-
**Squamous cell carcinoma**	**Overall**	23	15% (9% - 22%)	77.3%	<0.05
	**Country**				
	- USA	4	35% (15% - 57%)	91.5%	<0.05
	- Germany	4	29% (22% - 37%)	0%	0.54
	- USA/Australia	1	13% (4% - 38%)	-	-
	- Hungary	1	0% (0% - 23%)	-	-
	- Switzerland	3	8% (0% - 35%)	-	-
	- Italy	1	12% (5% - 27%)	-	-
	- Iran	2	9% (3% - 18%)	-	-
	- Korea	1	0%(0% - 32%)	-	-
	- Japan	4	10% (1% - 25%)	42.7%	0.16
	- Brazil	2	3%(0% -14%)	-	-
	**Continent**				
	- America	7	22% (9% - 39%)	88.1%	<0.05
	- Europe	9	18% (9% - 27%)	66%	<0.05
	- Asia	7	6% (0% - 17%)	61.7%	0.02
	**Sample type**				
	- Frozen section (Fr)	2	36%(28% - 44%)	-	-
	- Formalin-fixed paraffin-embedded (FFPE)	16	16% (9% - 23%)	74.9%	<0.05
	- Formalin-fixed paraffin-embedded / Frozen section (FFPE/Fr)	2	9% (0% - 26%)	-	-
	- Not reported (NR)	3	5% (0% - 24%)	-	-
**Basal cell carcinoma**	**Overall**	18	14% (7% - 22%)	82.6%	<0.05
	**Country**				
	- Germany	5	26% (14% - 40%)	71.2%	0.01
	- Hungary	1	0% (0% -28%)	-	-
	- France	1	0% (0% - 23%)	-	-
	- Switzerland	2	38% (27% - 49%)	-	-
	- Japan	4	4% (0% - 15%)	66.2%	0.03
	- Korea	1	0% (0% - 32%)	-	-
	- Brazil	3	24% (13% - 38%)	-	-
	- Iran	1	10% (5% - 20%)	-	-
	**Continent**				
	- America	3	24% (13% - 38%)	-	-
	- Europe	9	19% (8% - 32%)	79.4%	<0.05
	- Asia	6	5% (1% - 12%)	48.8%	0.08
	**Sample type**				
	- Frozen section (Fr)	2	31% (22% - 40%)	-	-
	- Formalin-fixed paraffin-embedded (FFPE)	12	14% (5% - 26%)	84.8%	<0.05
	- Formalin-fixed paraffin-embedded / Frozen section (FFPE/Fr)	2	0% (0% - 8%)	-	-
	- Not reported (NR)	2	11% (5% - 19%)	-	-
**Bowen’s disease**	**Overall**	6	21% (2% - 48%)	81.5%	-
	**Country**				
	- Germany	3	32% ( 18% - 48%)	-	-
	- Switzerland	1	25% (7% - 59%)	-	-
	- Japan	1	0% (0% - 10%)	-	-
	- Brazil	1	50% (15% - 85%)	-	-
	**Continent**				
	- America	1	50% (15% - 85%)	-	-
	- Europe	4	31% (18% - 45%)	0%	0.93
	- Asia	1	0%(0% - 10%)	-	-
	**Sample type**				
	- Frozen section (Fr)	1	50%(15% - 85%)	-	-
	- Formalin-fixed paraffin-embedded (FFPE)	4	17% (0% - 49%)	87.6%	<0.05
	- Not reported (NR)	1	25% (5% - 70%)	-	-
**Actinic keratosis**	**Overall**	6	6% (0% - 17%)	38.7%	0.148
	**Country**				
	- Germany	2	13% (3% - 27%)	-	-
	- Switzerland	1	0% (0% - 49%)	-	-
	- Japan	1	6% (2% - 16%)	-	-
	- Korea	1	0% (0% -32%)	-	-
	- Brazil	1	40% (12% - 77%)	-	-
	**Continent**				
	- America	1	40% (12% - 77%)	-	-
	- Europe	3	8% (0% - 25%)	-	-
	- Asia	2	3% (0% - 11%)	-	-
	**Sample type**				
	- Frozen section (Fr)	1	40% (12% - 77%)	-	-
	- Formalin-fixed paraffin-embedded (FFPE)	4	6% (0% - 16%)	32%	0.22
	- Not reported (NR)	1	0% (0% - 35%)	-	-
**Keratoacanthoma**	**Overall**	7	20% (0% - 51%)	91.6%	<0.05
	**Country**				
	- Germany	3	29%(20% - 39%)	-	-
	- Switzerland	1	0% (0% - 56%)	-	-
	- Brazil	1	100% (21% - 100%)	-	-
	- Sweden	1	36% (20% - 57%)	-	-
	- Korea	1	0% (0% - 51%)	-	-
	**Continent**				
	- America	1	100% (21% - 100%)	-	-
	- Europe	5	28%(20% - 38%)	0%	0.64
	- Asia	1	0% (0% - 4%)	-	-
	**Sample type**				
	- Frozen section (Fr)	1	100% (21% - 100%)	-	-
	- Formalin-fixed paraffin-embedded (FFPE)	5	15% (0% - 44%)	93.6%	<0.05
	- Not reported (NR)	1	43% (16% - 75%)	-	-
**Seborrheic keratosis**	**Overall**	5	10% (1% - 24%)	20%	0.287
	**Country**				
	- Germany	2	23% (10% - 39%)	-	-
	- Switzerland	1	0% (0% - 56%)	-	-
	- Japan	1	0% (0% - 43%)	-	-
	- Korea	1	0% (0% - 32%)	-	-
	**Continent**				
	- Europe	3	18%(6% - 34%)	-	-
	- Asia	2	0%(0% - 14%)	-	-
	**Sample type**				
	- Formalin-fixed paraffin-embedded (FFPE)	5	10% (1% - 24%)	20%	0.29

### Secondary Meta-Analyses: Non-MCC Skin Lesions and Normal Skin

#### Melanoma

**Figure 4:**
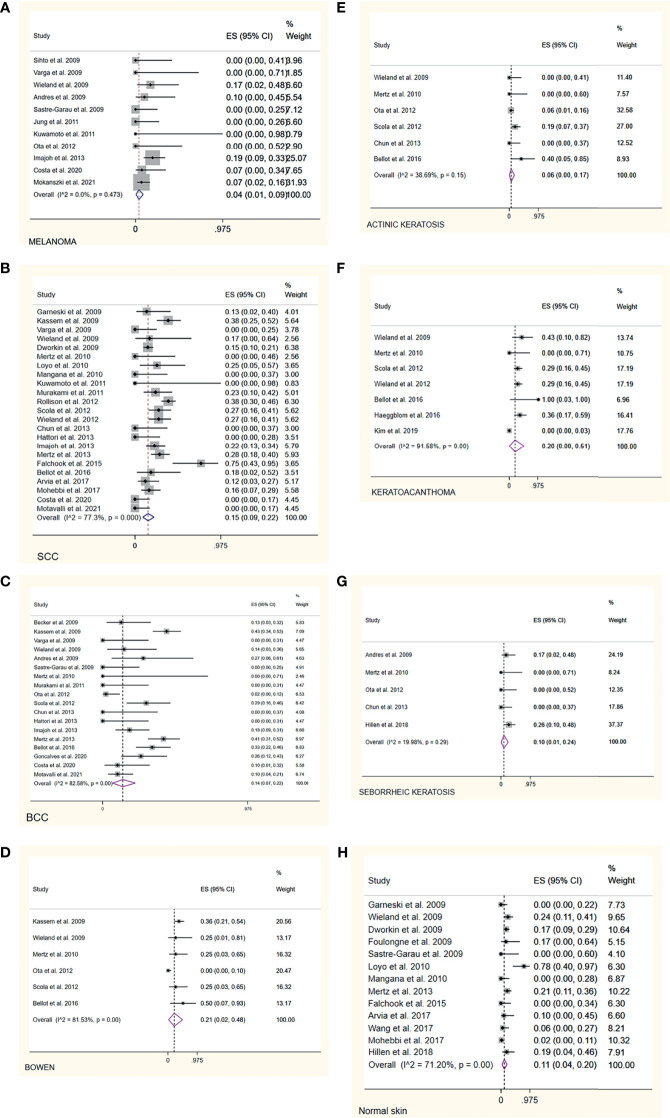
Forest plot illustrating the pooled prevalence rate of the MCPyV positivity in non-MCC skin lesions and normal skin. **(A)** melanoma; **(B)** squamous cell carcinoma; **(C)** basal cell carcinoma; **(D)** Bowen’s disease; **(E)** actinic keratosis; **(F)** keratoacanthoma; **(G)** seborrheic keratosis; **(H)** normal skin.

Eleven studies (11, 18, 19, 21, 22, 26, 39, 41, 46, 51, 60)investigated the prevalence rate of MCPyV+ in melanoma, the overall prevalence rate was 4% (95% CI = 1% - 9%, I^2^ = 0%, *P* = 0.473)(**Figure 4A**). In addition, subgroup analysis by country, continent, and sample type still showed significant heterogeneity (**Table 2** and **Figures S10–12**). The funnel plot, Egger’s test (*P* = 0.150), and Begg’s test (*P* = 0.080) detected no publication bias.

#### Squamous Cell Carcinoma

Twenty three studies (10, 13, 19, 21, 25, 29, 30, 35, 40–45, 47–49, 51–54, 56, 60) reported the prevalence rate of MCPyV+ in squamous cell carcinoma samples, with the overall prevalence rate was 15%(95% CI = 9% - 22%, I^2^ = 77.3%, *P*<0.05)(**Figure 4B**). The pooled prevalence rate remained similar in the stratified analysis, with statistically significant heterogeneity across all subgroups(**Table 2** and **Figures S13-15**). We discovered a significant difference in pooled MCPyV+ prevalence in squamous cell carcinoma in American studies 22%(95% CI = 9% - 39%) when compared to Asian studies 6%(95% CI = 0% - 17%), but not when compared to prevalence in Europe 18%(95% CI = 9% - 27%). The point estimates for the prevalence of MCPyV+ in squamous cell carcinoma in frozen section sample 36%(95% CI = 28% - 44%) was twice of the formalin-fixed paraffin-embedded sample. There was no evidence of publication bias as indicated by funnel plot analysis, Egger’s test(*P* = 0.133), and Begg’s test(*P* = 0.065).

#### Basal Cell Carcinoma

The 18 included studies (11, 16, 19, 21, 22, 29, 30, 40, 42, 44–46, 48, 51, 52, 54, 59, 60) reported the prevalence rate of the MCPyV+ in basal cell carcinoma, with the overall prevalence rate was 14%(95% CI = 7% - 22%, I^2^ = 82.58%, *P*<0.05)(**Figure 4C**). Stratification analysis showed increasing trends for American studies 24%(95% CI = 13% - 38%) and stable trends for European 19%(95% CI = 8% - 32%) and Asian studies 5%(95% CI = 1% - 12%). Frozen section samples 31%(95% CI = 22% - 40%) showed a higher prevalence rate than FFPE samples 14%(95% CI = 5% - 26%). While stratification analysis still showed significant heterogeneity(**Table 2** **and** **Figures S16-18**). According to the funnel plot, Egger’s test(*P* = 0.059), and Begg’s test(*P* = 0.075), there was no significant publication bias across the studies for either analysis.

#### Bowen’s Disease

Several studies (21, 42, 44, 46, 48, 54) investigated the prevalence rate of MCPyV+ in Bowen’s disease, with the pooled prevalence rate was 21%(95% CI = 2% - 48%, I^2^ = 81.53%, *P*<0.05)(**Figure 4D**). All subgroup analysis still showed significant heterogeneity (**Table 2** and **Figures S19-21**). In addition, there was an apparent lower prevalence in Asia than Americas(0% vs 50%). The funnel plot, Egger’s test(*P* = 0.257), and Begg’s test(*P* = 0.388) revealed no substantial publication bias.

#### Actinic Keratosis

The pooled analysis of six studies (21, 29, 44, 46, 48, 54) reporting the prevalence of MCPyV+ in actinic keratosis showed a prevalence rate of 6%(95% CI = 0% - 17%, I^2^ = 38.69%, *P* = 0.15)(**Figure 4E**). Results of the stratification analysis are shown in **Table 2** and **Figures S22-24**. Visual inspection of the funnel plot, Egger’s test(*P* = 0.899), and Begg’s test(*P* = 0.274), there was no evidence of significant publication bias.

#### Keratoacanthoma

According to seven publications (21, 44, 48, 49, 54, 55, 57) that examined the prevalence rate of MCPyV+ in keratoacanthoma, the pooled prevalence rate was 20%(95% CI = 0% - 51%), I^2^ = 91.58%, *P*<0.05)(**Figure 4F**). Stratified analysis showed statistically significant heterogeneity in all subgroups, although the pooled prevalence rate remained identical (**Table 2** and **Figures S25–27**). There was no evidence of substantial publication bias, as determined by visual inspection of the funnel plot, Egger’s test(*P* = 0.126), and Begg’s test(*P* = 0.301).

#### Seborrheic Keratosis

Five studies (22, 29, 37, 44, 46) were included in the analysis of the prevalence rate of MCPyV+ in seborrheic keratosis, with the overall prevalence rate was 10% (95% CI = 1% - 24%, I^2^ = 19.98%, *P* = 0.29)(**Figure 4G**). This pooled rate remained consistent in subgroup analysis, with statistically significant heterogeneity between subgroups (**Table 2** and **Figures S28-30**). According to the funnel plot analysis, Egger’s test(*P* = 0.105), and Begg’s test(*P* = 0.072) there was no evidence of publication bias.

#### Normal Skin

Based on data from 13 publications (10, 11, 13, 21, 24, 25, 34, 35, 37, 43, 52, 53, 56) the overall pooled estimate of the prevalence of MCPyV+ in normal skin was 11% (95% CI = 4% - 20%, I^2^ = 71.2%, *P*<0.05)(**Figure 4H**). Further stratification by country, continent, and sample type are shown in **Table 2** and **Figures S7-9**. In the USA, the American continent, and the FFPE study subgroups, heterogeneity remained significant. No publication bias was detected by funnel plot, Egger’s test (*P* = 0.967), or Begg’s test (*P* = 0.802).

## Discussion

Numerous factors contribute to the aetiology of non-MCC skin lesions, including UV exposure, immunosuppression, and ageing, which are also risk factors for the development of MCC (45, 53). Feng et al. (8) first discovered MCPyV as a human polyomavirus that reveals clonal integration in MCC. MCPyV showed that the viral genome was integrated into the host genome, disrupting the late region. In addition, a C-terminal truncated LT was expressed. The helicase activity of LT, which is required for viral DNA replication, was removed by this deletion (16). MCPyV infects the majority of people and, according to seroepidemiological studies, causes lifelong harmless chronic infection in healthy people (61–63). MCPyV is also regularly shed from the skin of healthy people, proving that it is a component of the human skin microbiome (64). Dermal fibroblast cells could be the natural host cell for replication of MCPyV in the human body, as the virus could be propagated in human dermal fibroblast cell cultures (65). The role of MCPyV in the development of MCC and the wide distribution of the virus in the body prompted researchers to investigate the prevalence of MCPyV in non-MCC skin lesions. Several studies have shown clonal integration of MCPyV in the non-MCC skin lesions. However, the prevalence of MCPyV in the MCC and non-MCC skin lesions is still controversial. Our study aimed to shed light on this matter.

To the best of our knowledge, this is the first systematic review and meta-analysis to provide comprehensive, up-to-date estimates of the association of MCPyV in MCC and non-MCC skin lesions. We identified a global pooled prevalence of 80% MCPyV+ among 1112 patients with MCC. This finding is consistent with a previous meta-analysis by Santos-Juanes et al. (66) which reported a prevalence of 79%. A geographic and sample type variation of MCPyV+ MCC has well been documented in a previous study. Data from the Americas and Europe show that nearly 80% of MCC cases are MCPyV+ (10, 67), while studies from Australia found that only 24% of cases are MCPyV+ (67). The lower prevalence of MCPyV+ in Australian studies compared to other continents may be due to the increased sun exposure in Australia, making a possible viral contribution less common and the possibility that a different and unknown strain of MCPyV is undetectable (10). In Asia, MCPyV+ is found in 76.9% to 88.5% of Japanese (29, 41, 45, 46, 48), 81.2% to 85.71% of Korean (29, 57), and 25% of Indian MCC patients (58). Several studies have shown that the MCPyV detection rate of DNA was greater in frozen samples than in FFPE tissue samples (12, 27). On the contrary, through subgroup analyses, we found no significant differences in the prevalence rate of MCPyV+ MCC among countries, continents, and different sample types (**Table 2**).

The discovery of MCPyV DNA in non-melanoma skin cancers(NMSCs) from immunocompromised people was the first observation linking MCPyV to non-MCC (15). MCPyV was later found in various non-MCC skin lesions and normal skin (**Table 1**). Recent studies showed that non-MCC skin lesions significantly have lower MCPyV DNA viral loads than in MCC. MCPyV DNA was significantly positive in non-melanoma skin cancer in immunosuppressed patients compared with non-immunosuppressed patients (38, 48, 68). Our meta-analytic study showed that the pooled prevalence rate of MCPyV+ in melanoma, SCC, BCC, Bowen’s disease, actinic keratosis, keratoacanthoma, seborrheic keratosis, and normal skin was 4%, 15%, 14%, 21%, 6%, 20%, 10%, and 11%, respectively (**Table 2**). The low prevalence rate of MCPyV in non-MCC skin lesions, which is similar or even lower to that in normal skin, suggests that MCPyV probably plays a minor role in the development of non-MCC skin lesions. Subgroup analysis by continent showed that trends were higher in the Americas for SCC, BCC, Bowen’s disease, actinic keratosis, and keratoacanthomas, with the corresponding rates being lower or relatively similar to the overall pooled prevalence in the Asian and European continents, respectively. In addition, we found that the detection rate for DNA extracted from frozen section samples was higher than for DNA extracted from FFPE samples, suggesting that degradation of DNA in FFPE tissues caused by formalin fixation makes PCR less sensitive (12, 20, 24, 27). The presence of MCPyV DNA in the skin and non-MCC skin lesions might not be a surprising phenomenon, as one would expect, because it is due to the ability of HPyVs to infect the skin and remain in a latent form that can be reactivated in states of profound immunosuppression (69, 70). MCPyV is a cutaneous microbe that is generally acquired in early childhood when it has the opportunity to integrate into the host genome of dermal fibroblast cells (65, 71). Regardless of these findings, it is apparent that the presence of MCPyV DNA alone is not sufficient to cause malignancy (38). Therefore, the oncogenic significance of MCPyV in non-MCC skin lesions is still debatable.

The limitations of our article also warrant considerations. First, because randomized trials are neither currently available nor likely to be conducted in the future, this meta-analysis relies on observational data. As a result, unmeasured biases in individual studies must be taken into consideration. Second, further assessment revealed that there were several sources of heterogeneity among the included studies: (1) heterogeneity of study population(age, gender, immune status, smoking and drinking habits, geographic differences, sun exposure, etc.), (2) the relatively small number of specimens examined may give a wrong view of the prevalence of MCPyV in specific samples, (3) methods performed to detect MCPyV viral load(i.e., primers selection, viral DNA copy number, etc.), and (4) PCR screening method (i.e., the quality of the samples, viral gene target selection, DNA extraction method, primer selection, PCR technique, false-positivity due to PCR contamination, etc.). To overcome these problems and convincingly determine MCPyV positivity, several multimodal approaches have recently been proposed, such as immunohistochemistry and PCR assay (IHC + PCR), fluorescence *in situ* hybridization(FISH) coupled with DNA hybridization chain reaction(HCR-DNA FISH), etc., which have been shown to be a highly sensitive approach to detect the viral genome in tissue samples (72, 73). Third, MCPyV may contribute to cancer onset through a “hit-and-run” mechanism (74, 75). Therefore, tumor samples from different stages should be examined because the virus has only transient effects in cellular transformation, as it can be silenced or its genome lost during cancer progression (76).

## Conclusion

Our results suggest a ubiquitous distribution of MCPyV in the skin with higher MCPyV positivity in MCC tumors, closely linking MCPyV as a putative etiologic agent to the carcinogenesis of MCC. However, the significantly lower prevalence rate of MCPyV+ in non-MCC skin lesions does not exclude a pathogenic association of this virus with the development of non-MCC skin lesions. Further large-scale studies using uniform viral genome detection methods are needed to determine the precise role of MCPyV in MCC pathogenesis and to define the significance of detecting viral DNA in non-MCC skin lesions.

## Data Availability Statement

The original contributions presented in the study are included in the article/**Supplementary Material**. Further inquiries can be directed to the corresponding authors.

## Author Contributions

WW, conceptualization, methodology, visualization, and writing—original draft preparation, formal analysis, investigation, writing— review and editing, and supervision. ZL, YQ, supervision and funding acquisition. WW, YL methodology and visualization. YL data curationand sample contribution. All authors contributed to the article and approved the submitted version.

## Funding

This research article was funded by the Science and Technology Support Program of Science and Technology Department of Sichuan Province (2020YFS0267), the Key Project Research and Invention Program of Science and Technology Department of Sichuan Province(2021YFS0245), the National Natural Science Foundation of China (81871574).

## Conflict of Interest

The authors declare that the research was conducted in the absence of any commercial or financial relationships that could be construed as a potential conflict of interest.

## Publisher’s Note

All claims expressed in this article are solely those of the authors and do not necessarily represent those of their affiliated organizations, or those of the publisher, the editors and the reviewers. Any product that may be evaluated in this article, or claim that may be made by its manufacturer, is not guaranteed or endorsed by the publisher.
