# Supplementary material for: Prevalence of Merkel Cell Polyomavirus in Normal and Lesional Skin: A Systematic Review and Meta-Analysis
Source: Front Oncol. 2022 Mar 22;12:868781. doi: 10.3389/fonc.2022.868781 (PMC8980839; doi:10.3389/fonc.2022.868781)
Supplement: Supplementary file 1 [file DataSheet_1.docx]

Supplementary information to:

**Prevalence of Merkel cell polyomavirus in normal and lesional skin: a systematic review and meta-analysis**

***Supplementary Materials***

List of supplementary materials:

1. **Table S1**: Quality assessment via Newcastle Ottawa scale and recall bias risk.
2. **Figure S1:** Sensitivity analyses for studies on the association between MCV and MCC.
3. **Figure S2**: Funnel plot for studies on the association between MCV and MCC.
4. **Figure S3**: Funnel plot for studies on the association between MCV and MCC with trim and fill method.
5. **Figure S4**: Forest plot of pooled prevalence rate of the MCV in MCC patients based on country.
6. **Figure S5**: Forest plot of pooled prevalence rate of the MCV in MCC patients based on continent.
7. **Figure S6**: Forest plot of pooled prevalence rate of the MCV in MCC patients based on sample type.
8. **Figure S7**: Forest plot of pooled prevalence rate of the MCV in normal skin based on country.
9. **Figure S8**: Forest plot of pooled prevalence rate of the MCV in normal skin based on country.
10. **Figure S9**: Forest plot of pooled prevalence rate of the MCV in normal skin based on sample type.
11. **Figure S10**: Forest plot of pooled prevalence rate of the MCV in melanoma based on country.
12. **Figure S11**: Forest plot of pooled prevalence rate of the MCV in melanoma based on continent.
13. **Figure S12**: Forest plot of pooled prevalence rate of the MCV in melanoma based on sample type.
14. **Figure S13**: Forest plot of pooled prevalence rate of the MCV in squamous cell carcinoma based on country.
15. **Figure S14**: Forest plot of pooled prevalence rate of the MCV in squamous cell carcinoma based on continent.
16. **Figure S15**: Forest plot of pooled prevalence rate of the MCV in squamous cell carcinoma based on sample type.
17. **Figure S16**: Forest plot of pooled prevalence rate of the MCV in basal cell carcinoma based on country.
18. **Figure S17**: Forest plot of pooled prevalence rate of the MCV in basal cell carcinoma based on continent.
19. **Figure S18**: Forest plot of pooled prevalence rate of the MCV in basal cell carcinoma based on sample type.
20. **Figure S19**: Forest plot of pooled prevalence rate of the MCV in Bowen’s disease based on country.
21. **Figure S20**: Forest plot of pooled prevalence rate of the MCV in Bowen’s disease based on continent.
22. **Figure S21**: Forest plot of pooled prevalence rate of the MCV in Bowen’s disease based on sample type.
23. **Figure S22**: Forest plot of pooled prevalence rate of the MCV in actinic keratosis based on country.
24. **Figure S23**: Forest plot of pooled prevalence rate of the MCV in actinic keratosis based on continent.
25. **Figure S24**: Forest plot of pooled prevalence rate of the MCV in actinic keratosis based on sample type.
26. **Figure S25**: Forest plot of pooled prevalence rate of the MCV in keratoacanthoma based on country.
27. **Figure S26**: Forest plot of pooled prevalence rate of the MCV in keratoacanthoma based on continent.
28. **Figure S27**: Forest plot of pooled prevalence rate of the MCV in keratoacanthoma based on sample type.
29. **Figure S28**: Forest plot of pooled prevalence rate of the MCV in seborrheic keratosis based on country.
30. **Figure S29**: Forest plot of pooled prevalence rate of the MCV in seborrheic keratosis based on continent.
31. **Figure S30**: Forest plot of pooled prevalence rate of the MCV in seborrheic keratosis based on sample type.

**Table S1. Quality assessment** via Newcastle Ottawa scale and recall bias risk.

| **Study** | **Selection** | | | | **Comparability** | **Outcome** | | | **Total** |
| --- | --- | --- | --- | --- | --- | --- | --- | --- | --- |
|  |  |  |  |  |  |  |  |  |  |
|  |  |  |  |  |  |  |  |  |  |
| Feng et al. 2008 ^[8]^ | 1 | 1 | 0 | 1 | 1 | 1 | 1 | 1 | 7 |
| Kassem et al. 2008 ^[15]^ | 1 | 1 | 1 | 1 | 1 | 1 | 0 | 1 | 7 |
| Becker et al. 2009 ^[16]^ | 1 | 1 | 0 | 1 | 1 | 1 | 1 | 1 | 7 |
| Garneski et al. 2009 ^[10]^ | 1 | 1 | 0 | 1 | 1 | 1 | 1 | 1 | 7 |
| Helmbold et al. 2009 ^[17]^ | 1 | 1 | 1 | 1 | 1 | 0 | 1 | 1 | 7 |
| Kassem et al. 2009 ^[18]^ | 1 | 1 | 0 | 0 | 0 | 1 | 0 | 1 | 4 |
| Sihto et al. 2009 ^[19]^ | 1 | 1 | 0 | 1 | 1 | 1 | 1 | 1 | 7 |
| Varga et al. 2009 ^[20]^ | 1 | 1 | 0 | 1 | 1 | 1 | 1 | 1 | 7 |
| Touze et al. 2009 ^[21]^ | 1 | 1 | 0 | 1 | 1 | 1 | 1 | 1 | 7 |
| Wieland et al. 2009 ^[22]^ | 1 | 1 | 0 | 1 | 1 | 1 | 1 | 1 | 7 |
| Andres et al. 2009 ^[23]^ | 1 | 1 | 0 | 1 | 1 | 1 | 1 | 1 | 7 |
| Bhatia et al. 2009 ^[24]^ | 1 | 1 | 0 | 1 | 1 | 1 | 1 | 1 | 7 |
| Dworkin et al. 2009 ^[43]^ | 1 | 1 | 0 | 0 | 0 | 1 | 0 | 1 | 4 |
| Foulongne et al. 2009 ^[25]^ | 1 | 1 | 0 | 1 | 1 | 1 | 1 | 1 | 7 |
| Sastre-Garau et al. 2009 ^[11]^ | 1 | 1 | 0 | 1 | 1 | 1 | 1 | 1 | 7 |
| Mertz et al. 2010 ^[44]^ | 1 | 1 | 0 | 0 | 0 | 1 | 0 | 1 | 4 |
| Loyo et al. 2010 ^[13]^ | 1 | 1 | 0 | 1 | 1 | 1 | 1 | 1 | 7 |
| Mangana et al. 2010 ^[26]^ | 1 | 1 | 0 | 1 | 1 | 1 | 1 | 1 | 7 |
| Jung et al. 2011 ^[27]^ | 1 | 1 | 0 | 1 | 1 | 1 | 1 | 1 | 7 |
| Kuwamoto et al. 2011 ^[28]^ | 1 | 1 | 0 | 1 | 0 | 1 | 1 | 1 | 6 |
| Murakami et al. 2011 ^[45]^ | 1 | 1 | 0 | 0 | 0 | 1 | 0 | 1 | 4 |
| Martel-Jantin et al. 2012 ^[29]^ | 1 | 1 | 1 | 1 | 1 | 1 | 0 | 1 | 7 |
| Ota et al. 2012 ^[46]^ | 1 | 1 | 0 | 0 | 0 | 1 | 0 | 1 | 4 |
| Rodig et al. 2012 ^[30]^ | 1 | 1 | 0 | 1 | 1 | 1 | 1 | 1 | 7 |
| Rollison et al. 2012 ^[47]^ | 1 | 1 | 0 | 0 | 0 | 1 | 0 | 1 | 4 |
| Scola et al. 2012 ^[48]^ | 1 | 1 | 0 | 0 | 0 | 1 | 0 | 1 | 4 |
| Wieland et al. 2012 ^[49]^ | 1 | 1 | 0 | 0 | 0 | 1 | 0 | 1 | 4 |
| Iwasaki et al. 2013 ^[50]^ | 1 | 1 | 0 | 0 | 0 | 1 | 0 | 1 | 4 |
| Chun et al. 2013 ^[31]^ | 1 | 1 | 0 | 1 | 1 | 1 | 1 | 1 | 7 |
| Hattori et al. 2013 ^[32]^ | 1 | 1 | 0 | 1 | 1 | 1 | 1 | 1 | 7 |
| Fukumoto et al. 2013 ^[33]^ | 1 | 1 | 0 | 1 | 1 | 1 | 1 | 1 | 7 |
| Imajoh et al. 2013 ^[51]^ | 1 | 1 | 0 | 0 | 0 | 1 | 0 | 1 | 4 |
| Mertz et al. 2013 ^[52]^ | 1 | 1 | 0 | 0 | 0 | 1 | 0 | 1 | 4 |
| Leroux-Kozal et al. 2015 ^[34]^ | 1 | 1 | 0 | 1 | 1 | 1 | 1 | 1 | 7 |
| Falchook et al. 2015 ^[53]^ | 1 | 1 | 0 | 0 | 0 | 1 | 0 | 1 | 4 |
| Bellot et al. 2016 ^[54^] | 1 | 1 | 0 | 0 | 0 | 1 | 0 | 1 | 4 |
| Haeggblom et al. 2016 ^[55]^ | 1 | 1 | 0 | 0 | 0 | 1 | 0 | 1 | 4 |
| Alvarez-Arguelles et al. 2017 ^[35]^ | 1 | 1 | 0 | 1 | 1 | 1 | 1 | 1 | 7 |
| Arvia et al. 2017 ^[56]^ | 1 | 1 | 0 | 0 | 0 | 1 | 0 | 1 | 4 |
| Wang et al. 2017 ^[36]^ | 1 | 1 | 0 | 1 | 1 | 1 | 1 | 1 | 7 |
| Mohebbi et al. 2017 ^[37]^ | 1 | 1 | 0 | 1 | 1 | 1 | 1 | 1 | 7 |
| Kervarrec et al. 2018 ^[38]^ | 1 | 1 | 1 | 1 | 0 | 1 | 1 | 1 | 7 |
| Hillen et al. 2018 ^[39]^ | 1 | 1 | 0 | 1 | 1 | 1 | 1 | 1 | 7 |
| Kim et al. 2019 ^[57]^ | 1 | 1 | 0 | 0 | 0 | 1 | 0 | 1 | 4 |
| Rekhi et al. 2019 ^[58]^ | 1 | 1 | 0 | 0 | 0 | 1 | 0 | 1 | 4 |
| Neto et al. 2019 ^[40]^ | 1 | 1 | 1 | 1 | 0 | 1 | 1 | 1 | 7 |
| Goncalves et al. 2020 ^[59]^ | 1 | 1 | 0 | 0 | 0 | 1 | 0 | 1 | 4 |
| Costa et al. 2020 ^[60]^ | 1 | 1 | 0 | 0 | 0 | 1 | 0 | 1 | 4 |
| Mokanszki et al. 2021 ^[41]^ | 1 | 1 | 0 | 1 | 1 | 1 | 1 | 1 | 7 |
| Motavalli et al. 2021 ^[42]^ | 1 | 1 | 1 | 0 | 1 | 1 | 1 | 1 | 7 |

Note. 1. Representativeness of the exposed cohort; 2. selection of the unexposed cohort; 3. ascertainment of exposure; 4. evidence that the outcome of interest was not present at baseline; 5. comparability of cohorts based on design or analysis; 6. ascertainment of exposure; 7. same method of ascertainment for cases and controls?; 8. non-response rate.

**Figure S1.** Sensitivity analyses for studies on the association between MCV and MCC.

**Figure S2**. Funnel plot for studies on the association between MCV and MCC

**Figure S3**. Funnel plot for studies on the association between MCV and MCC with trim and fill method.

**Figure S4**. Forest plot of pooled prevalence rate of the MCV in MCC patients based on country.

**Figure S5**. Forest plot of pooled prevalence rate of the MCV in MCC patients based on continent.

**Figure S6**. Forest plot of pooled prevalence rate of the MCV in MCC patients based on sample type.

**Figure S7**. Forest plot of pooled prevalence rate of the MCV in normal skin based on country.

**Figure S8**. Forest plot of pooled prevalence rate of the MCV in normal skin based on continent.

**Figure S9**. Forest plot of pooled prevalence rate of the MCV in normal skin based on sample type.

**Figure S10**. Forest plot of pooled prevalence rate of the MCV in melanoma based on country.

**Figure S11**. Forest plot of pooled prevalence rate of the MCV in melanoma based on continent.

**Figure S12**. Forest plot of pooled prevalence rate of the MCV in melanoma based on sample type.

**Figure S13**: Forest plot of pooled prevalence rate of the MCV in squamous cell carcinoma based on country.

**Figure S14**: Forest plot of pooled prevalence rate of the MCV in squamous cell carcinoma based on continent.

**Figure S15**: Forest plot of pooled prevalence rate of the MCV in squamous cell carcinoma based on sample type.

**Figure S16**. Forest plot of pooled prevalence rate of the MCV in basal cell carcinoma based on country.

**Figure S17**. Forest plot of pooled prevalence rate of the MCV in basal cell carcinoma based on continent.

**Figure S18**. Forest plot of pooled prevalence rate of the MCV in basal cell carcinoma based on sample type.

**Figure S19**. Forest plot of pooled prevalence rate of the MCV in Bowen’s disease based on country.

**Figure S20**. Forest plot of pooled prevalence rate of the MCV in Bowen’s disease based on continent.

**Figure S21**. Forest plot of pooled prevalence rate of the MCV in Bowen’s disease based on sample type.

**Figure S22.** Forest plot of pooled prevalence rate of the MCV in actinic keratosis based on country.

**Figure S23**. Forest plot of pooled prevalence rate of the MCV in actinic keratosis based on continent.

**Figure S24**. Forest plot of pooled prevalence rate of the MCV in actinic keratosis based on sample type.

**Figure S25**. Forest plot of pooled prevalence rate of the MCV in keratoacanthoma based on country.

**Figure S26**. Forest plot of pooled prevalence rate of the MCV in keratoacanthoma based on continent.

**Figure S27**. Forest plot of pooled prevalence rate of the MCV in keratoacanthoma based on sample type.

**Figure S28**. Forest plot of pooled prevalence rate of the MCV in seborrheic keratosis based on country.

**Figure S29**. Forest plot of pooled prevalence rate of the MCV in seborrheic keratosis based on continent.

**Figure S30**. Forest plot of pooled prevalence rate of the MCV in seborrheic keratosis based on sample type.
